# Determinants of Neonatal Jaundice among Neonates Admitted to Neonatal Intensive Care Unit in Public General Hospitals of Central Zone, Tigray, Northern Ethiopia, 2019: a Case-Control Study

**DOI:** 10.1155/2020/4743974

**Published:** 2020-10-21

**Authors:** Guesh Gebreayezgi Asefa, Teklay Guesh Gebrewahid, Hailemariam Nuguse, Mengistu Welday Gebremichael, Merhawi Birhane, Kidane Zereabruk, Teklewoini Mariye Zemicheal, Abrha Hailay, Woldu Aberhe Abrha, Surafel Aregawi Hadera, Areaya Gebreegzabiher Hailu, Brhane Hagos Beyene, Ebud Ayele Dagnazgi, Fsaha Gebretsadkan Tekulu, FissahaTekulu Welay

**Affiliations:** ^1^Department of Biostatistics and Epidemiology, College of Health Sciences, School of Public health, Aksum University, Axum, Ethiopia; ^2^Department of Clinical Midwifery, College of Health Sciences, Mekelle University, Mekelle, Ethiopia; ^3^School of Nursing, College of Health Sciences, Aksum University, Axum, Ethiopia; ^4^Department of Midwifery, College of Health Sciences, Aksum University, Axum, Ethiopia; ^5^Department Public health, Gamby Medical and Business College, Ethiopia; ^6^Department of Midwifery, College of Health Sciences, Dilla University, Dilla, Ethiopia; ^7^Department of Human Nutrition, College of Health Sciences, School of Public Health, Aksum University, Axum, Ethiopia; ^8^Department of Reproductive Health, College of Health Sciences, School of Public Health, Aksum University, Axum, Ethiopia; ^9^Department of Midwifery, College of Medicine and Health Sciences, Adigrat University, Adigrat, Ethiopia

## Abstract

**Background:**

Neonatal jaundice is common a clinical problem worldwide. Globally, every year, about 1.1 million babies develop severe hyperbilirubinemia with or without bilirubin encephalopathy and the vast majority reside in sub-Saharan Africa and South Asia. Strategies and information on determinants of neonatal jaundice in sub-Saharan Africa are limited. So, investigating determinant factors of neonatal jaundice has paramount importance in mitigating jaundice-related neonatal morbidity and mortality. *Methodology*. Hospital-based unmatched case-control study was conducted by reviewing medical charts of 272 neonates in public general hospitals of the central zone of Tigray, northern Ethiopia. The sample size was calculated using Epi Info version 7.2.2.12, and participants were selected using a simple random sampling technique. One year medical record documents were included in the study. Data were collected through a data extraction format looking on the cards. Data were entered to the EpiData Manager version 4.4.2.1 and exported to SPSS version 20 for analysis. Descriptive and multivariate analysis was performed. Binary logistic regression was used to test the association between independent and dependent variables. Variables at *p* value less than 0.25 in bivariate analysis were entered to a multivariable analysis to identify the determinant factors of jaundice. The level of significance was declared at *p* value <0.05.

**Results:**

A total of 272 neonatal medical charts were included. Obstetric complication (AOR: 5.77; 95% CI: 1.85-17.98), low birth weight (AOR: 4.27; 95% CI:1.58-11.56), birth asphyxia (AOR: 4.83; 95% CI: 1.617-14.4), RH-incompatibility (AOR: 5.45; 95% CI: 1.58-18.74), breastfeeding (AOR: 6.11; 95% CI: 1.71-21.90) and polycythemia (AOR: 7.32; 95% CI: 2.51-21.311) were the determinants of neonatal jaundice.

**Conclusion:**

Obstetric complication, low birth weight, birth asphyxia, RH-incompatibility, breastfeeding, and polycythemia were among the determinants of neonatal jaundice. Hence, early prevention and timely treatment of neonatal jaundice are important since it was a cause of long-term complication and death in neonates.

## 1. Introduction

Jaundice is derived from the French word *Juan* which means yellow [1]. Neonatal jaundice (NNJ) is the yellow discoloration of the skin, sclera, and mucosa caused by excess accumulation of bilirubin in the tissue and plasma (serum bilirubin level should be in excess 7 mg/dl). It occurs in up to 60-80% of preterm and term as well as 10% of breastfeeding neonates [2]. The bilirubin level in neonates is much higher than in adults because the life span of the erythrocytes is relatively short and the capacity for bilirubin elimination is lower than in adults; however, hyperbilirubinemia, or jaundice, is a life-threatening disorder in newborns [3, 4].

Neonatal jaundice is a common clinical problem worldwide. Globally, every year, about 1.1 million babies would develop severe hyperbilirubinemia with or without bilirubin encephalopathy, and the majority resides in sub-Saharan Africa and South Asia. In Nigeria, it is 100 times more than in developed countries [5, 6]. The burden was highest in low and middle income countries of subsaharan Africa and South Asia [7]. The global burden of neonatal juandice reported that the African region has the highest incidence of severe neonatal jaundice per 1000 live births (667.8 to 738.5) followed by the Southeast Asian (251.3 to 473.2) and Americas and European regions 4.4 and 3.7 respectively [8]. Ethiopia is one of the top ten countries with jaundice-related neonatal mortality [9]. A study done at Gonder University showed that jaundice was among the causes of neonatal admission and deaths [10].

Severe neonatal jaundice leads to acute bilirubin encephalopathy or kernicterus with a significant risk of neonatal mortality and long-term neurologic damage such as cerebral palsy, sensory neural hearing loss, intellectual difficulties, or gross developmental delays [11]. It is estimated for 75% hospitalization which needs medical concern and hospital readmission in newborns [11]. It results in brain encephalopathy which requires close attention, evualation and treatment. It also increases the economic and social burden on the patient's families and societies. There are well-developed systems to identify, investigate, and manage the problem in developed countries, but studies and development are still required to address the problem in poor countries [8].

A study in developed countries reveals that blood incompatibilities are the main causes of neonatal jaundice, whereas prematurity, low birth weight, G6PD deficiency, infection, and traditional practice such as herbal consumption and application of dusting powder were causes of NNJ in developing countries [12].

Understanding of determinant factors of jaundice is crucial to prevent and control the problem. Investigating the factors among the cases is also important to prevent the devastating morbidity and mortality. An evidence-based strategy is needed for prevention, early detection, and treatment. As far as our knowledge is concerned, information on determinants of neonatal jaundice, preventive programs, and uniform practice guidelines, including developmental assessment and surveillance of neonates with jaundice were not available at the health care delivery of the study area. Therefore, identifying the determinant factors of neonatal jaundice has paramount importance in mitigating jaundice-related neonatal morbidity and mortality. The result of this study would help in formulating measures of improving prevention, early detection, and management of neonatal jaundice. Adequate study on factors responsible for the occurrence of NNJ is still lacking in the study area. Therefore, preventing and controlling jaundice desires an understanding of the determinant factors in decisive manner.

## 2. Materials and Methods

### 2.1. Aim of the Study

The objective of this study is to identify the determinants of neonatal jaundice among neonates admitted to NICU in the public general hospitals of the central zone of Tigray, Ethiopia.

### 2.2. Study Design

In this study, a hospital-based unmatched case-control study design was used.

### 2.3. Study Setting and Period

The study was conducted in public general hospitals of the central zone of Tigray. The central zone had an estimated total population of 1,283,388 according to the Ethiopian Central Statistics report of 2007. The zone has three public general hospitals, one referral hospital, eight district hospitals, and fifty-seven health centers. The public general hospitals are Saint Marry Axum, Adwa, and Abyi Adi hospitals. The study was carried out from August 20 to September 20, 2019.

### 2.4. Population

#### 2.4.1. Source Population

*Cases*. All medical record documents of jaundiced neonates admitted to NICU in the public general hospitals of the central zone.

*Controls*. All medical record documents of neonates' without jaundice admitted to NICU in the public general hospitals of the central zone.

#### 2.4.2. Study Population

*Cases*. All medical record documents of jaundiced neonates who were admitted to NICU in the public general hospitals of the central zone within the last one year.

*Control.* All medical record documents of neonates without jaundice who were admitted to NICU in the public general hospitals of the central zone within the last one year.

### 2.5. Sample Size Determination

The required sample size was determined using Epi Info version 7.2.2.12 from the previous study conducted in southern India on maternal and neonatal determinants of jaundice with the assumptions and parameters of power 80%, 95% level of certainty, percent of cases with prim parity of 67.8%, percent of control with prim parity of 49.2%, and OR 2.17 and 2 : 1 ratio with regard to prim parity [13]. The estimated sample size was cases = 91 and controls = 181. Hereby, the actual sample for this study was 272.

### 2.6. Sampling Technique and Procedure

The study was conducted in the central zone, and all the public general hospitals of the central zone, Adwa, ST Marry Axum, and Abyi Adi hospitals, were included. Medical record documents of cases and controls were used for data collection. Cases and controls were selected by simple random sampling method. The sample size was allocated to each hospital proportionally.

### 2.7. Data Collection Tool and Procedures

The data were collected using extraction format and review of medical record documents. One year medical record documents were included in the study. The extract formats were developed by reviewing previous similar studies that consist of all the variables that can meet the objective of the study. It includes sociodemographic, obstetric, maternal, and neonatal factors. Pretest was done on 5% of the sample on both cases and controls in Suhul Shire General Hospital before ten days of actual data collection. Necessary corrections were done based on the information obtained from the pretest result.

### 2.8. Data Quality Control

Three data collectors (midwifery professionals) and three supervisors were involved in the data collection. The principal investigator and coordinators have attended the activities on a daily base to give clarification and support for data collectors. Training was given for the data collectors and supervisors. In the training session, the data collectors have been oriented on the objective of the study and how to collect data. The supervisor had assessed the performance of data collectors and correct any problem encountered together with the principal investigator. The collected data were reviewed and checked for completeness by the principal investigator.

### 2.9. Data Processing and Analysis

Data was entered using the EpiData Manager and exported to SPSS version 20. Further cleaning and recoding were done before the analysis. Bivariate and multivariable logistic regression analysis was used at a 95% confidence interval for the existence of the association.

All variables with *p* value <0.25 in bivariate analysis were entered in the multivariable logistic regression. The strength of the association between dependent and independent variables was measured using odds ratio at 95% confidence interval (CI), and *p* value <0.05 was used to determine the level of statistical significance. Model fitness was checked by the Hosmer and Lemeshow test. Multicollinearity was checked by S.E, tolerance, and VIF. The result was presented using numerical value, texts, percentages, tables and frequencies, mean, median, and standard deviation.

#### 2.10. Operational Definition

*Low APGAR score*. A neonate was classified in the APGAR score if it scored ≤4.

*Polycythemia*. A neonate whose RBC was ≥65 mg/dl was categorized under polycythemia.

## 3. Results

### 3.1. Sociodemographic Characteristics of the Neonates and Their Mother

A total of 272 with 91 cases and 181 controls were included in the study. The median age of the neonates (controls) at admission was 2 (±2) days with a maximum of 19 days and a minimum of two hours, whereas for the cases was 2 (±2) days with a maximum of 20 days and a minimum of 6 hours. The mean age of the mothers of the controls and cases was 28.91 (±6.497) and 29.25 (±6.764), respectively (see Table 1).

### 3.2. Obstetric Characteristics of the Last Pregnancy

One hundred thirteen (62.4%) of the control and 51 (56%) of the cases were born from multipara mothers. More than 3/4 (79.1%) of the controls and more than 2/3 (69%) of the cases were RH positive. One hundred two (56.4%) of the controls and 60 (65.9%) of the cases were delivered at the hospital, whereas fifteen (8.3%) of the controls and six (6.6%) of the cases were delivered at home. Fifty-eight (32%) of the controls and 20 (22%) of the cases were delivered at a health center. The remaining neonates were delivered on the vehicle transporting to a health institution. (see Table 2).

### 3.3. Neonatal Characteristics

The mean amount of hemoglobin for the controls was 16 (±3.428), and those of cases were 17.84 (4.0 ± 19). The mean amount of total bilirubin level of controls was 10.66 (±5.98), and the median amount of direct bilirubin level of cases was 1.22 (±2).

**Figure 1:**
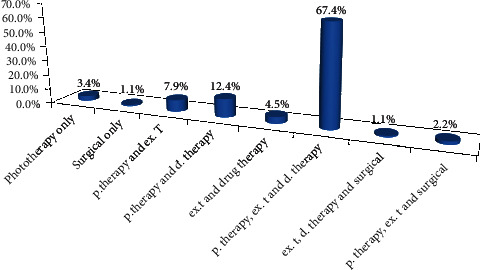
Treatment type given for the cases of jaundice. p.therapy: phototherapy, d.therapy: drug therapy, ex.t: blood exchange transfusion.

According to the type of treatment given to cases of jaundice, 2/3 of the cases were treated with phototherapy, exchange transfusion, and drug therapy together (see Figure 1).

Nearly 3/4 of the cases had a bilirubin level of <15 mg/dl (see Table 3).

**Figure 2:**
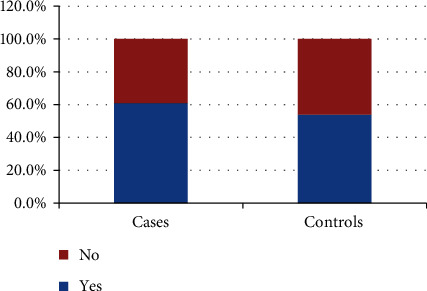
ABO compatibility between mothers and babies.

Regarding the ABO compatibility between mothers and babies, 60.9% of the cases and 53.9% of the controls encountered ABO incompatibility (see Figure 2).

Looking at the chi-square test, there is no significant statistical difference in the odds of developing jaundice between the cases and controls. At a 5% level of significance, from the data, there is sufficient evidence to conclude that the distribution of ABO incompatibility was the same on cases and controls of jaundice (*p* value = 0.272).

### 3.4. Determinants of Neonatal Jaundice

The bivariate logistic regression analysis showed that maternal blood group, obstetric complication, mode of delivery, neonatal birth weight, breastfeeding, neonatal sepsis, birth asphyxia, cephalohematoma, neonatal blood group, RH incompatibility, polycythemia, and hepatitis B status had a *p* value <0.25 and were eligible for multiple logistic regression. However, the statistically significant determinants of neonatal jaundice in the multivariable analysis were obstetric complication, low birth weight, birth asphyxia, “B” blood type of the neonate, RH incompatibility, polycythemia, breastfeeding and maternal “O” blood group with *p* < 0.05.

The odds ratio of obstetric complication was 5.8 times higher among jaundiced neonates as compared to the controls (AOR: 5.77 at 95% CI: 1.85-17.98). The odds of maternal blood group “O” mother was 90% less to develop jaundice compared to blood type A among the cases (AOR: 0.10 at 95% CI: 0.022-0.38). The odds of low birth weight was 4.3 times more among jaundiced neonates compared to the controls (AOR: 4.27 at 95% CI: 1.579-11.555). The odds of birth asphyxia was 4.8 times among jaundiced compared to the controls (AOR: 4.83 at 95% CI: 1.617-14.395). Neonates with RH incompatibility were 5.5 times at high risk for jaundice compared to the controls (AOR: 5.45 at 95% CI: 1.583-18.737). The odds of breastfeeding was 6 times higher among the cases compared to the controls (AOR: 6.11 at 95% CI: 1.707-21.886). Polycythemia (hematocrit > 65%) was more frequent in neonates with jaundice as compared to neonates without jaundice (AOR: 7.32 (2.51-21.31)). Neonatal “B” blood type had a negative association with neonatal jaundice (AOR: 0.22 at 95% CI: 0.076-0.602). The odds of cephalohematoma was 4.9 times more among the cases compared to the controls (AOR: 4.86 at 95% CI: 1.173-20.131) (see Table 4).

## 4. Discussion

This study was aimed at assessing the determinants of neonatal jaundice among neonates admitted to the neonatal intensive care unit in public general hospitals of the central zone Tigray, Ethiopia.

The finding of this study showed that RH incompatibility, low birth weight, breastfeeding, polycythemia, obstetric complication, birth asphyxia, B blood type of the neonate, and maternal O blood group were the determinants of neonatal jaundice among the neonates who were admitted to the NICU at public general hospitals of the central zone.

Neonatal jaundice was positively associated with RH incompatibility compared to those without incompatibility. The finding of this study was similar to a study conducted in the Mekelle public hospitals and TASH (Addis Ababa) [14, 15]. It was also consistent with the findings in Efftu (Ghana) and Canada [16, 17]. Low birth weight was positively associated with the development of neonatal jaundice. This was in agreement with the study conducted in Effutu Municipality of Ghana, Central Hospital of Nigeria, Government Medical Colleges of South India [13, 16, 18, 19]. This might be due to similarity in gestational age among study populations.

The result of this study revealed that neonatal jaundice was positively associated with breastfeeding. This was consistent with the study done in Addis Ababa [15], Southeast Nigeria [20], India [18], and Southern Nepal [21]. This association is due to the fact that breastfeeding leads to substantial elevation of bilirubin levels during the first few days of life by inhibiting conjugation of bilirubin due to the existence of nonesterified free fatty acid called pregnanediol [22]. However, it was contradicting with the study result in India [13]. The disparity may be related to the sample size difference.

In this study, jaundice was positively associated to obstetric complication. This was correspondent with the study report of Mekelle [15], Harare [23], and India [18]. Birth asphyxia was higher in the jaundiced neonates compared to the controls. This was supported by the case-control study conducted in India [13, 18]. Neonates with polycythemia were high risk to develop jaundice compared to normal hematocrit. This was similar to a study conducted in Nehru Hospital (India) and Shaare Medical Center of Jerusalem [24–26]. Maternal blood group O was associated with neonatal jaundice. This was supported by the study in Mekelle [15] and Jerusalem [26]. ABO incompatibility occurs when the mother is O type while the neonates are type A, B, and AB. Maternal blood group O with anti-A and anti-B crosses the placenta might hurt neonates with blood group A, B and AB.

In fact, this study reported LBW was positively associated with jaundice (*p* value = 0.004). This might be due to many (68.4%) of LBW neonates in this study were male babies. So, this may be associated with G6PD as this disease affects males lonely.

### 4.1. Limitation

The diagnosis of jaundice by health care providers widely varies and depends both on observer attention and on infants' characteristics.

## 5. Conclusion

Neonatal jaundice was a common cause of neonatal morbidity and mortality. The major determinants of neonatal jaundice in this study were RH incompatibility, obstetric complication, asphyxia, low birth weight, polycythemia, and breastfeeding. Therefore, identifying the determinants will enable to develop the preventive measures and to identify the high-risk cases. So, early prevention and timely treatment of neonatal jaundice are important to prevent long-term complications and death in neonates.

## Figures and Tables

**Table 1:** Sociodemographic characteristics of the neonates and their mothers in public general hospitals of central zone, Tigray, 2019.

Variables	Category	Controls (*n* = 181)	Cases (*n* = 91)	Total (*n* = 272)
Age of mothers	Mean (±SD)	28.91 (±6.497)	29.25 (±6.764)	
Residence	Urban	119 (65.7%)	57 (62.6%)	176 (64.7%)
	Rural	62 (34.3%)	34 (37.4%)	96 (35.3%)
Ethnicity	Tigray	162 (89.5%)	85 (93.4%)	247 (90.8%)
	Amara	19 (10.5%)	6 (6.6%)	25 (9.2%)
Sex of neonates	Male	116 (64.1%)	57 (62.6%)	173 (63.6%)
	Female	65 (35.9%)	34 (37.4%)	99 (36.4%)
Age of neonates at admission in days	Median (±IQR)	2 (±2)	2 (±2)	
Maternal age	≤19	4 (2.2%)	8 (8.8%)	12 (4.4%)
	20-34	141 (77.9%)	60 (65.9)	201 (73.9%)
	35-49	36 (19.9%)	23 (25.3%)	59 (21.7%)

**Table 2:** Obstetric characteristics of the last pregnancy in public general hospitals of central zone, Tigray, 2019.

Variable	Category	Control	Case	Total
Gravidity	Prim	37 (20.4%)	23 (25.5%)	60 (22.06%)
	Multi	120 (66.3%)	58 (63.7%)	178 (65.44%)
	Grand	24 (13.3%)	10 (11%)	34 (12.5%)
Gestational age	Term	123 (68%)	62 (68.1%)	185 (68.01%)
	Preterm	36 (19.9%)	21 (23.1%)	57 (20.96%)
	Postterm	22 (12.2%)	8 (8.8%)	30 (11.03%)
Type of pregnancy	Single	175 (96.7%)	83 (91.2%)	258 (94.9%)
	Twin	6 (3.3%)	8 (8.8%)	14 (5.1%)
Abortion	Yes	45 (24.9%)	22 (24.2%)	67 (24.6%)
	No	136 (75.1%)	69 (75.8%)	205 (76.4%)
Maternal blood group	A	63 (34.8%)	36 (39.6%)	99 (36.4%)
	B	39 (21.6%)	18 (19.8%)	57 (21%)
	AB	20 (11%)	9 (9.9%)	29 (11%)
	O	17 (9.4%)	24 (26.4)	41 (15.1%)
	Unknown	42 (23.2%)	4 (4.4%)	46 (17%)
Maternal RH factor	+Ve	110 (60.8%)	60 (69%)	170 (75.2%)
	-Ve	29 (16%)	27 (31%)	56 (24.8%)
	Unknown	42 (23.2%)	4 (4.4%)	46 (18%)
Obstetric complication	Yes	17 (9.4%)	20 (22%)	37 (13.6%)
	No	164 (90.6%)	71 (78%)	235 (86.4)
Onset of labour	Spontaneous	154 (85.1)	71 (78%)	225 (82.7%)
	Induced	27 (14.9%)	20 (22%)	47 (17.3%)
Duration of labour	Normal	162 (89.5%)	75 (82.4%)	247 (90.8%)
	Prolonged	19 (10.5%)	16 (17.6%)	35 (9.2%)
Mode of delivery	Instrumental	19 (10.5%)	14 (15.4%)	33 (12%)
	c/s	19 (10.5%)	17 (18.7%)	36 (13%)
	SVD	143 (79%)	60 (65.9%)	203 (75%)

c/s: cesarean section, SVD: spontaneous vaginal delivery.

**Table 3:** Neonatal characteristics in public general hospitals of central zone, Tigray, 2019.

Variable	Category	Control (*n* = 181)	Case (*n* = 91)	Total
Sex	Male	116 (64.1%)	57 (62.6%)	173 (63.3%)
	Female	65 (35.9%)	34 (37.4%)	99 (36.4%)
APGAR measured	Yes	111 (61.3%)	60 (65.9%)	171 (62.9%)
	No	70 (38.7%)	31 (34.1%)	101 (37.1%)
Five minute APGAR	Low	79 (71.2%)	45 (75%)	124 (45.6%)
	Normal	32 (28.8%)	15 (25%)	47 (17.3%)
Birth weight	LBW	27 (14.9%)	23 (25.3%)	50 (18.4%)
	Macrocosmic	10 (5.5%)	8 (8.8%)	18 (6.6%)
	Normal	144 (79.6%)	60 (65.9%)	204 (75%)
Feeding option	Not feeding	24 (13.3%)	11 (12.1%)	35 (12.9%)
	Breastfeeding	126 (69.6%)	74 (81.3%)	200 (73.5%)
	Formula feeding	31 (17.1%)	6 (6.6%)	37 (13.6%)
Birth asphyxia	Yes	40 (22.1%)	20 (22%)	60 (22%)
	Unknown	30 (16.6%)	27 (29.7%)	57 (21%)
	No	111 (61.3%)	44 (48.4%)	155 (57%)
Neonatal RH factor	+Ve	102 (56.4%)	59 (64.8%)	161 (70.6%)
	−Ve	35 (19.3%)	32 (35.2%)	67 (29.4%)
	Unknown	44 (24.3%)		
Random blood sugar	Hypoglycemia	32 (17.7%)	12 (13.2%)	44 (16.2%)
	Hyperglycemia	14 (7.7%)	6 (1.1%)	15 (5.5%)
	Normal	135 (74.6%)	78 (85.7%)	213 (78.3%)
Outcome	Improved	137 (75.7%)	48 (52.7%)	185 (68.1%)
	Dead	23 (12.7%)	19 (20.9%)	42 (15.4%)
	Referred	21 (11.6%)	24 (26.4%)	45 (16.5%)
Bilirubin level in mg/dl	<15	_	69 (75.8%)	_
	15-19.9	_	17 (18.7%)	_
	20-24.9	_	3 (3.3%)	_
	25-30	_	2 (2.2%)	_

LBW: low birth weight.

**Table 4:** Determinants of neonatal jaundice at neonatal intensive care units in public general hospitals of central zone, Tigray, 2019.

Variables	Category	Case (*n* = 91)	Control (*n* = 181)	COR (95% CI)	AOR (95% CI)
Obstetric complication	Yes	20 (22%)	17 (9.4%)	2.72 (1.34-5.49)	5.77 (1.85-17.98) ^∗^*p* = 0.002
	No	71 (78%)	164 (90.6%)	1	1
Birth weight	LBW	23 (25.3%)	27 (14.9%)	2.04 (1.09-3.85)	4.27 (1.58-11.56) ^∗^*p* = 0.004
	Normal	60 (65.9%)	144 (79.6%)	1	1
	Macrocosmic	8 (8.8%)	10 (5.5%)	1.4 (0.61-2)	4.56 (0.396-52.14)
Birth asphyxia	Yes	20 (22%)	40 (22.1%)	1.26 (0.67-2.39)	4.83 (1.62-14.4) ^∗^*p* = 005
	Unknown	27 (29.7%)	30 (16.6%)	2.27 (1.21-4.25)	2.36 (0.86-6.48)
	No	44 (48.4%)	111 (61.3%)	1	1
Cephalohematoma	Yes	16 (17.6%)	12 (6.6)	3.0 (1.36-6.66)	4.86 (1.173-20.131)
	No	75 (82.4%)	169 (93.4%)	1	1
RH incompatibility	Yes	16 (17.6%)	7 (5.1%)	3.96 (1.56-10.07)	5.45 (1.58-18.74) ^∗^*p* = .007
	No	75 (82.4%)	130 (94.9%)	1	1
Breastfeeding	Not feeding	11 (12.1%)	24 (13.3%)	2.37 (0.77-7.32)	1.52 (0.29-7.93)
	Breast feed	74 (81.3%)	126 (69.6%)	3.03 (1.21-7.62)	6.11 (1.71-21.89)^∗^*p* = .005
	Formula feed	6 (6.6%)	31 (17.1%)	1	1
Polycythemia	Yes	21 (23.1%)	13 (7.2%)	3.88 (1.84-8.17)	7.32 (2.51-21.31)^∗^*p* = .000
	No	70 (76.9%)	168 (92.8%)	1	1
Hepatitis B status	Unknown	24 (26.4%)	90 (49.7%)	0.34 (0.19-0.59)	0.15 (0.06-0.37) ^∗^
	Reactive	7 (7.7%)	15 (8.3%)	0.59 (0.23-1.54)	0.35 (0.03-1.47)
	Nonreactive	60 (65.9%)	76 (42%)	1	1
Neonatal sepsis	Yes	43 (47.3%)	58 (32%)	1.9 (1.1-3.18)	1.74 (0.79-3.82)
	No	48 (52.7%)	123 (68%)	1	1
Mode of delivery	Instrumental	14 (15.4%)	19 (10.5%)	1.76 (0.83-3.73)	0.97 (0.20-4.65)
	c/s	17 (18.7%)	19 (10.5%)	2.13 (1.04-4.38)	0.75 (0.25-2.25)
	SVD	60 (65.9%)	143 (79%)	1	1
Maternal blood	Unknown	24 (26.4%)	17 (9.4%)	2.47 (1.17-5.20)	3.26 (0.98-10.74)^∗^
	B	18 (19.8%)	39 (21.6%)	15 (4.5-49.2)	0.36 (0.01-2.06)
	AB	9 (9.9%)	20 (11%)	4.7 (1.3-17.9)	0.47 (0.07-3.05)
	O	5 (5.5%)	42 (23.2%)	0.21 (0.08-0.57)	0.10 (0.02-0.38)^∗^
	A	36 (39.6%)	63 (34.8%)	1	1
Blood group neonate	Unknown	5 (5.5%)	46 (25.4%)	0.12 (0.04-0.34)	4.76 (0.52-43.39)
	O	24 (26.4%)	25 (13.8)	1.09 (0.53-2.21)	1.12 (0.41-3.03)
	AB	9 (9.9%)	10 (5.5%)	0.3 (0.15-0.61)	0.57 (0.14-2.28)
	B	15 (16.5%)	57 (31.5%)	0.3 (0.15-0.61)	0.22 (0.08-0.61) ^∗^*p* = .000
	A	38 (41.8%)	43 (23.8%)	1	1

N.B ^∗^ indicates the determinant of neonatal jaundice at *p* value <0.05. LBW: low birth weight, c/s: cesarean section, SVD: spontaneous vaginal delivery.

## Data Availability

The dataset analyzed in the current study is available from the corresponding author on reasonable request.

## Abbreviations

AOR: Adjusted odds ratio
C/D: Cesarean delivery
EBF: Exclusive breastfeeding
EDHS: Ethiopian Demographic and Health Survey
LBW: Low birth weighs
LMICs: Low- and middle-income countries
mg/dl: Milligram per deciliter
NNJ: Neonatal jaundice
NICU: Neonatal intensive care unit
PROM: Premature rapture of membrane
SPSS: Statically Package software for Social Science.
